# Distinct virulence of the microsporidian parasite in honey bees competing habitat

**DOI:** 10.3389/fcimb.2025.1524197

**Published:** 2025-02-17

**Authors:** Xiuxiu Wei, Qiang Huang

**Affiliations:** Honeybee Research Institute, Jiangxi Agricultural University, Nanchang, China

**Keywords:** *Nosema ceranae*, host switch, habitat competition, selection, mortality

## Abstract

In natural ecosystems, parasites often infect multiple host species, particularly when hosts share habitats, facilitating host-to-host transmission and altering traditional host-parasite coevolution dynamics. This study examines the microsporidian parasite *Nosema ceranae* in Eastern honey bees (*Apis cerana*) and Western honey bees (*Apis mellifera*), assessing its virulence and proliferation dynamics. Using inoculation experiments, we measured bee mortality and parasite spore loads to infer virulence and proliferation. Additionally, time-series transcriptome analysis of both bees and parasites provide insights into host-pathogen interactions. The results reveal that *N. ceranae* produces more spores with lower mortality in *A. mellifera* but causes higher mortality with lower spore production in *A. cerana*. The parasite also suppresses host gene expression, with stronger suppression observed in *A. cerana*. These findings suggest that *N. ceranae* is adapted for low virulence and high proliferation in *A. mellifera* but exhibits high virulence and limited proliferation in *A. cerana*. This study highlights the evolution of distinct trade-offs between virulence and proliferation in a multi-host system, offering valuable insights into parasite-host dynamics and their ecological implications.

## 1 Introduction

Host mortality typically assesses parasite virulence, which is not static but evolves depending on host ecology (Gowler et al., 2023). Most evolutionary virulence theories connect the trade-off between the parasite and host fitness. For example, high parasite proliferation increases the transmission to other individuals. However, high proliferation may kill the host rapidly and reduce parasite transmission (de Roode et al., 2008). Thus, balanced transmission and virulence were expected in co-evolved host-parasite (Acevedo et al., 2019).

In natural ecosystems, parasites migrate and explore alternative host organisms. The phylogenetic relationship and proximity of the host species influence the success of host shifting, where closely related species most likely share parasites (Engelstädter and Fortuna, 2019). For example, *Nosema ceranae* infects both the Asian honey bee *Apis cerana* and the European honey bee *Apis mellifera* (Fries et al., 1996; Higes et al., 2007). The infection starts from ingesting spore-contaminated nectar. The spores germinate in the midgut lumen and inject the sporoplasm into the epithelial cells through the polar tube (Gisder et al., 2011). The infected honey bees show suppressed apoptosis (Higes et al., 2013), immature aging (Paris et al., 2018), shortened life span (Eiri et al., 2015), and impaired flight (Gage et al., 2018).

In Asia, the two honey bee species compete for habitats and shelter resources, and the prevalence of the parasite *N. ceranae* has been over 70% in both bee species (Li et al., 2012; Yang et al., 2013; Jack et al., 2016). Thus, the chance is high that the parasite switches between the two honey bee species back and forth (Graystock et al., 2015). *N. ceranae* infection changes the global gene expression in both European and Asia honey bees (Holt et al., 2013; Fan et al., 2022). The parasite infection causes colony failure in European honey bees (Higes et al., 2008; Botías et al., 2013). Comparatively, its virulence in Asia honey bees is unclear. Previously, we found that the host habitat sharing increases the parasite gene flow (Ke et al., 2022). In this follow-up study, we use two honey bee species and a microsporidian parasite to investigate how host habitat sharing shapes the parasite virulence. We find a distinct trade-off between virulence and proliferation in the two closely related honey bees in a shared habitat.

## 2 Materials and methods

### 2.1 Ethical statement

The honey bees *Apis mellifera* and *Apis cerana* are neither protected nor endangered species. Ethical approval is not required for this study.

### 2.2 Hosts and parasite sources

We designed a two-by-two factorial experiment to study parasite virulence and proliferation in two honey bee species. We used two parasite sources P_Acer_ (Parasite spores purified from the honey bee *Apis cerana*) and P_Amel_ (Parasite spores purified from the honey bee *Apis mellifera*), as well as two host species H_Acer_ (Host honey bee *Apis cerana*) and H_Amel_ (Host honey bees *Apis mellifera*) (**Supplementary Table S1**). We combined bees from different hives for the inoculation, and uninfected bees (H_Acer_ and H_Amel_) as controls for this multi-host-parasite experiment. The honey bee colonies are maintained in the experimental apiary at Jiangxi Agricultural University.

### 2.3 Parasite isolation, inoculation, and RNA extraction

Three hundred honey bees of each *A. mellifera* and *A. cerana* were captured near the hive entrance using an insect net. The midgut was dissected and homogenized to isolate *N. ceranae* spores that were further purified using the Percoll gradient (Chen et al., 2013). The spores were counted under the light microscope using a hemacytometer. The sealed brood frames from three hives of H_Amel_ and H_Acer_ were kept in an incubator to collect newly emerged bees (35°C, 75% humidity).

The newly emerged honey bee workers (H_Acer_ and H_Amel_) were individually inoculated with 2 µL of sugar solution with 10^5^ spores. Additional freshly emerged honey bees (< 24 h after emerging) were each fed 2 µL of sugar solution without spores as the uninfected control group. One hundred fifty honey bees were inoculated in each treatment group, and the cohorts were divided into 3 rearing cups (50 bees per cup) in an incubator (35°C, 75% humidity) (3 replicates, **Supplementary Table S1**) to investigate the general bee response and parasite proliferation. During the experiment, sucrose (50% w/w) was provided *ad libitum* as the only food. In each rearing cup, three bees were collected at 24 h intervals from 1 to 5 dpi (day post-inoculation) for RNA-seq. The remaining bees were dissected to count spores at 14 dpi.

### 2.4 RNA extraction and library preparation

As the parasite infects the epithelial cell in the midgut, we dissected midgut tissue for RNA-seq. Three bees per cup per day were dissected and pooled for RNA extraction using Trizol. The library was prepared using the NEBNext Ultra RNA Kit. In total, 90 RNA libraries (5 days * 6 treatments * 3 replicates) were sequenced on the Illumina NovaSeq 6000 platform.

### 2.5 Bioinformatics and statistics

The quality of RNA reads (150bp, paired-end) was first viewed using Fastqc (Andrews, 2010) and trimmed using the Seqtk package with default parameters (Li, 2022). The processed reads were aligned to *N. ceranae* (Version Ncer 3.0), *A. cerana* (version CC1.0), and *A. mellifera* (version Hav3.1) genome, respectively, with the Hisat2 package with default parameters (Kim et al., 2015; Diao et al., 2018; Wallberg et al., 2019; Huang et al., 2021). The read count per gene was retrieved using bedtools (Quinlan and Hall, 2010). The within-group dispersion was calculated from the three replicates to determine the significantly regulated genes with edgeR package and adjusted for FDR (false discovery rate) (Robinson et al., 2010). The genes with FDR < 0.05 were defined as significantly regulated ones. Gene Ontology (GO) terms were retrieved using EggNOG-mapper, and the enrichment analysis was performed using the TopGo package with an adjusted weighted ks test (Robinson et al., 2010; Alexa and Rahnenfuhrer, 2021). Bee survival was analyzed using the Kaplan-Meier estimate in the survival package, adjusted for FDR (R Core Team, 2013; Therneau, 2022). The variance of the spore load among the treatment groups was analyzed with the Wilcoxon rank test, and the p values were adjusted with FDR to reduce false positives. The impact of the spore source and day on the gene expression was analyzed using ANONA, where the day and parasite source were fixed factors, and the replicates were random factors. The number of up and down-regulated genes was analyzed using Pearson’s Chi-squared test.

## 3 Results

### 3.1 The parasites show distinct virulence in honey bees

**Figure 1:**
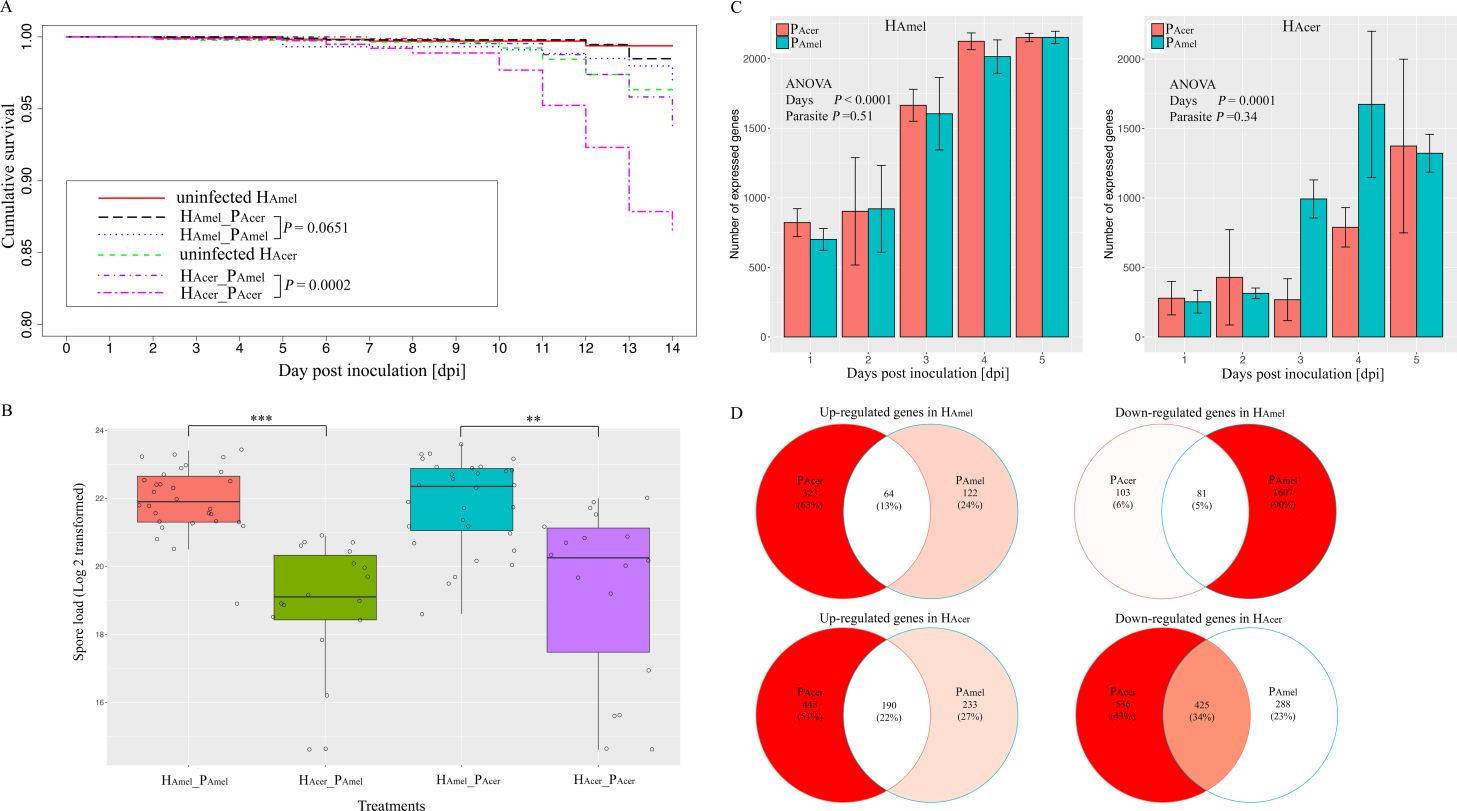
Transmission and virulence of the parasites and the bee responses towards the parasite in a two-parasite and two-host system. **(A)** Cumulative survival of the honey bees. The impact of the parasite source on the survival of bees was minor. The P_Acer_ caused substantially higher mortality than P_Amel_ in H_Acer_. **(B)** parasite proliferation variance in two honey bee species. The parasite produced more spores in H_Amel_ than H_Acer_. Additionally, the impacts of the parasite sources on the spore load were minor in either host. **(C)** The number of expressed parasite genes in two hosts. Fewer parasite genes were expressed in *A*. *cerana* than *A*. *mellifera*, irrespective of the parasite sources. **(D)** Venn diagram of the shared and unique regulated host genes responding to the two parasite isolates. Overall, the bee genes were down-regulated by the infection, and a subset of genes responded to both parasite sources. ** indicates the significance level at *P* < 0.01; *** indicates the significance level at *P* < 0.001. The error bar indicates the standard deviation.

The uninfected H_Amel_ shows the highest survival (99.4% survival), followed by H_Amel_ infected with P_Acer_ (H_Amel__P_Acer_, 98.5% survival) and the H_Amel__P_Amel_ group (96.9% survival). The bees in the H_Acer__P_Acer_ group show the lowest survival (86.6% survival). The parasite source (P_Amel_ and P_Acer_) shows a minor impact on the survival of H_Amel_ (H_Amel__P_Acer_ vs H_Amel__P_Amel_, Kaplan-Meier test, *P* > 0.05) (**Figure 1A**, **Supplementary Table S2**). Comparatively, the parasite P_Acer_ causes significantly higher mortality in H_Acer_ than P_Amel_ (Coxph test, *P* < 0.001). The uninfected H_Amel_ survives substantially better than the uninfected H_Acer_ (Coxph test, *P* < 0.01). Overall, the parasites cause a higher mortality in H_Acer_ than in H_Amel_. Additionally, we normalize the mortality variance between the two honey bee species using uninfected_H_Amel_ and uninfected_H_Acer_. Again, the parasites cause higher mortality in H_Acer_ than H_Amel_ (*P* < 0.001, **Supplementary Figure S1**).

### 3.2 The parasites show substantial proliferation variance

The spores are not found in the uninfected bees. The spore load is not evenly distributed among the four infected honey bee groups (Kruskal-Wallis test, χ^2^ = 43.1, df=3, *P* < 0.0001, **Figure 1B**). The parasite produces more spores in H_Amel_ than H_Acer_, when infected by either P_Amel_ or P_Acer_ (Wilcoxon rank sum test, df=1, *P* < 0.001) (**Supplementary Table S3**). Thus, host species substantially impact the parasite proliferation (F=32.3, df=2, ANOVA, *P* < 0.0001).

### 3.3 Higher parasite gene expression profile in H_Amel_ than H_Acer_

To investigate the parasite gene expression profile, we quantify the parasite transcriptome in the two host species (**Figure 1C**). H_Acer_ shows a stronger tendency to suppress the parasite gene expression than H_Amel_ (F=77.1, df=1, ANOVA, *P* < 0.0001). A significantly lower number of up-regulated genes are observed in P_Amel_ than P_Acer_ when infecting the H_Amel_ (Pearson’s Chi-squared test, χ^2^ = 20.8, df=2, *P* < 0.0001). The highest variance was at four dpi, and the number of up-regulated genes is threefold higher in P_Acer_ (36 genes) than in P_Amel_ (12 genes) in H_Amel_ (**Supplementary Table S4**). Thus, the parasites express a higher number of genes and transcript levels in H_Amel_ than in H_Acer_.

### 3.4 Host response variance toward the infection

**Figure 2:**
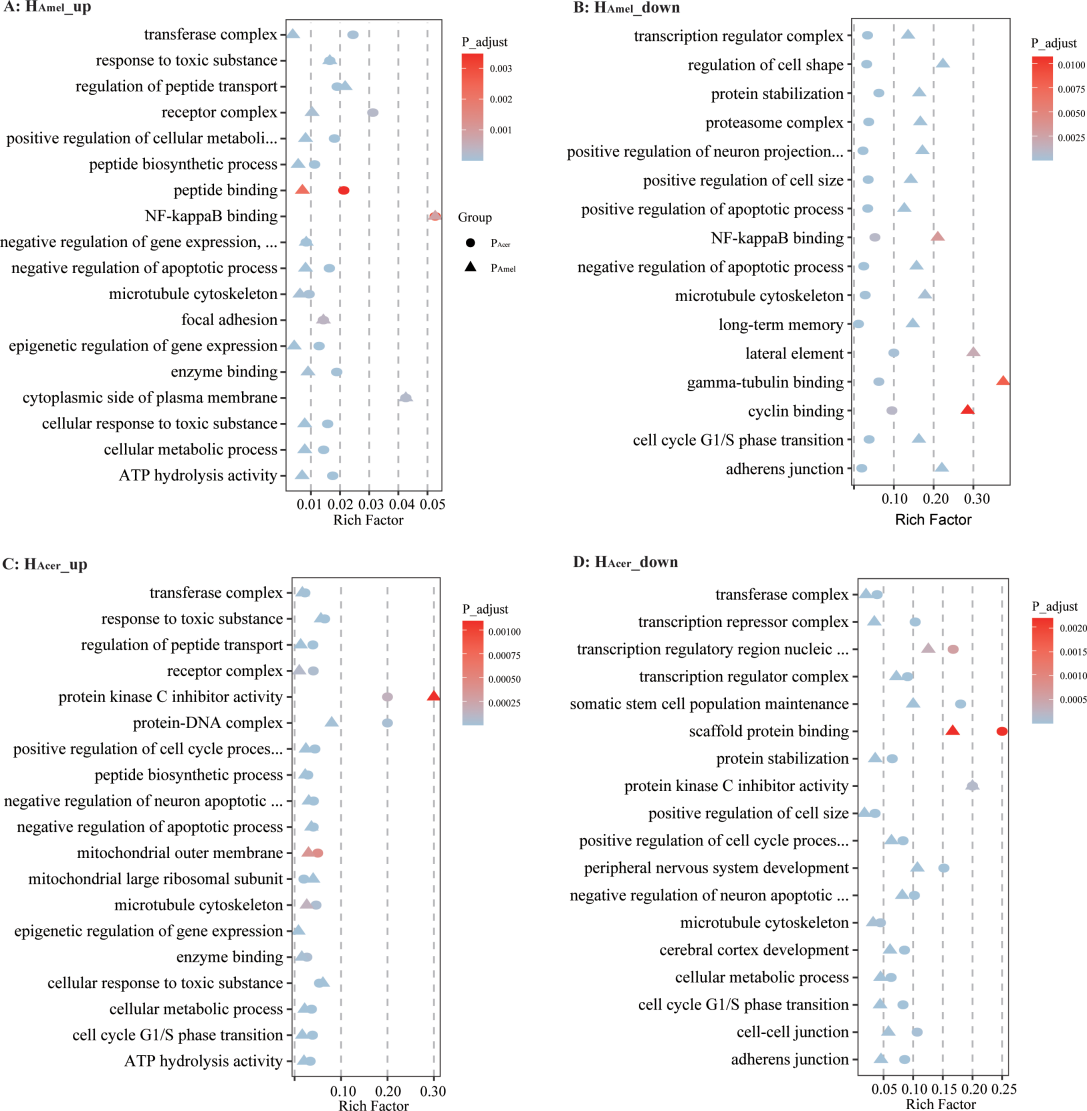
Bubble chart for GO enrichment of differentially expressed genes. The intersection of up-regulated genes at five time points in H_Amel_ infected with P_Amel_ and P_Acer_ **(A)**. The intersection of down-regulated genes at five time points in H_Amel_ **(B)**. Up-regulated genes at five time points in H_Acer_ **(C)** and down-regulated genes at five time points in H_Acer_ **(D)**. Upon parasitic infection, the honey bee enzyme binding and metabolism-related genes were up-regulated, while genes related to the cell cycle and transcriptional regulation were suppressed.

To infer how strong is the impact of the infection on the host, the honey bee transcriptomes responding to each of the two parasite sources are quantified as well. We find the expression of bee (H_Amel_ and H_Acer_) genes is suppressed, where more down-regulated genes than up-regulated ones when infected by either P_Amel_ or P_Acer_ (**Table 1**, Pearson’s Chi-squared test, χ^2^ = 51.0, df=4, *P* < 0.001). Comparatively, more genes were regulated by the infection in H_Acer_ than H_Amel_. The bee hosts share a common subset of genes that respond to infection (**Figure 1D**). The immune response toward the infection is small in H_Amel_, reflected by only two down-regulated immune genes in Toll pathway involved in pathogen recognition (LOC412536) and melanization (LOC406115). We found both up and down regulated immune genes in H_Acer_. Particularly, the Toll pathway is generally suppressed, including the genes involved in parasite recognition PGRP (APICC_00292), signal transport SPZ (APICC_01852), and antimicrobial peptides defensin and lysozyme (APICC_08301, APICC_03572, APICC_08272). These suppressed genes have previously been confirmed using qPCR (Ke et al., 2022). A few genes are continuously down-regulated in H_Amel_, reflecting genes inhibited by the infection (**Supplementary Figure S2**). GO enrichment analysis indicates that apoptosis regulation (GO:0043066, FDR< 0.0001), cell cycle phase transition (GO:0044843, FDR < 0.0001), and a few other biological functions are altered in the infected honey bee genes (**Figure 2**).

**Table 1:** The number of significantly regulated honey bee genes between infected and uninfected bees.

	H_Amel_											H_Acer_										
	1 dpi		2 dpi		3 dpi		4 dpi		5 dpi		P	1 dpi		2 dpi		3 dpi		4 dpi		5 dpi		P
	U	D	U	D	U	D	U	D	U	D		U	D	U	D	U	D	U	D	U	D	
P_Acer_	38	60	32	117	30	101	24	130	88	93	< 0.001	143	239	213	394	85	115	325	353	70	196	< 0.001
P_Amel_	28	121	32	219	23	213	27	265	105	1320	< 0.001	164	189	84	149	67	173	136	156	87	248	< 0.001

To quantify the impact of the parasite sources on the host species, the gene expression of the infected host was compared with the uninfected ones. The number of down-regulated honey bee genes was substantially larger than the up-regulated ones. U indicates the number of up-regulated genes; D indicates the number of down-regulated genes.

## 4 Discussion

A static environment favors decreased genetic diversity. In contrast, a fluctuating environment favors a high genetic diversity (Abdul-Rahman et al., 2021). The two bee species in Asia compete for the habitat, and the parasite can shift between them. To adapt to both bees, the parasite may favor a large gene pool to survive. Indeed, a higher diversity in the sympatric population than in the allopatric population was observed (Ke et al., 2022). Parasites show elevated genetic diversity by infecting diverse host populations, suggesting host diversity shapes parasite diversity (Ekroth et al., 2021).

Previous studies suggest that *N. ceranae* causes 40% ~ 90% of bee mortality, and the spore load is at 10^6^ levels in H_Amel_ two weeks post-inoculation (Higes et al., 2007; Paxton et al., 2007; Martín-Hernández et al., 2011; Suwannapong et al., 2011; Eiri et al., 2015). In our data, low mortality (4.1%) is observed in H_Amel_, and slightly higher mortality (13.4%) is observed in H_Acer_. The parasite shows low virulence in the primary host when the alternative host species are less common (Manzoli et al., 2018). Historically, the parasite is first described in *A. cerana* and does not necessarily indicate that *A. cerana* is the primary host (Fries et al., 1996). Subsequently, the parasite is identified in *A. mellifera* (Higes et al., 2007). The anthropogenic-driven contact enhanced the gene flow of the parasites (Pelin et al., 2015). If *A. cerana* is the primary host, a balanced transmission and virulence are expected between H_Acer_ and P_Acer_. In our data, the parasite shows high virulence and low spore load in H_Acer_, which do not follow the conventional host-parasite evolution. Thus, additional studies are needed to investigate whether H_Amel_ or H_Acer_ is the primary host of *N. ceranae*.

*N. ceranae* infection causes global gene expression changes in H_Amel_ and H_Acer_ (Holt et al., 2013; Fan et al., 2022). A few studies suggest the Toll pathway is the primary immune response to the *N. ceranae* infection (Huang and Evans, 2016; Li et al., 2017; Ke et al., 2022). In our data, we find the infection caused minor immune stress in H_Amel_. Comparatively, the infection suppresses the Toll pathway from pathway recognition to the antimicrobial peptides in H_Acer_. Additionally, the infection strongly regulates the transcripts, and more genes are altered by the infection, suggesting intense stress in H_Acer_. Remarkably, the infection causes higher mortality in Asia honey bees, which has been overlooked for decades. Lipid metabolism is important for microsporidians to establish infection (El Alaoui et al., 2001; Jeon, 2021). In our data, the up-regulated parasite genes are enriched in lipid metabolism, which might be necessary for *N. ceranae* to establish infection. The apoptosis pathway is also enriched in regulated honey bee genes, confirming apoptosis is an essential defense mechanism in bees against *N. ceranae* infection (Higes et al., 2013; Kurze et al., 2015). In bumblebees, the microsporidian parasite *Nosema bombi* shows distinctive virulence toward hosts in a sympatric population (Rutrecht and Brown, 2009). In *Daphnia*, the microsporidian shows reduced infection intensity with increased geographic distance (Ebert, 1994). In our case, bee genetics and co-evolutionary status may shape the distinct trade-offs between virulence and proliferation in *N. ceranae*. Future studies to identify the genome diversification of *N. ceranae* may help to determine its primary host. Additional gene functional studies help to understand the host-parasite co-evolution in this multi-host system, also as target genes for this parasite control.

## 5 Conclusions

The microsporidian parasite *N. ceranae* evolves a balanced virulence and transmission with the honey bee *A. mellifera* in Asia. Comparatively, the parasite shows high virulence and low transmission in the honey bee *A. cerana*, supported by the time series transcripts. Thus, additional study is needed to investigate whether *A. cerana* or *A. mellifera* is the primary host of this microsporidian parasite.

## Data Availability

The datasets presented in this study can be found in online repositories. The names of the repository/repositories and accession number(s) can be found in the article/**Supplementary Material**. The sequencing reads are available in NCBI Bio-project PRJNA822678 and PRJNA784016.
