# Supplementary material for: Distinct virulence of the microsporidian parasite in honey bees competing habitat
Source: Front Cell Infect Microbiol. 2025 Feb 17;15:1524197. doi: 10.3389/fcimb.2025.1524197 (PMC11873089; doi:10.3389/fcimb.2025.1524197)
Supplement: Supplementary file 1 [file Table1.docx]

Supplementary Material for

**Distinct virulence of the microsporidian parasite in honey bees competing habitat**

Xiuxiu Wei and Qiang Huang*

Honeybee Research Institute, Jiangxi Agricultural University, Zhimin Ave. 1101, Nanchang, 330045, China.

***** For correspondence, Email: [qiang-huang@live.com](mailto:qiang-huang@live.com)


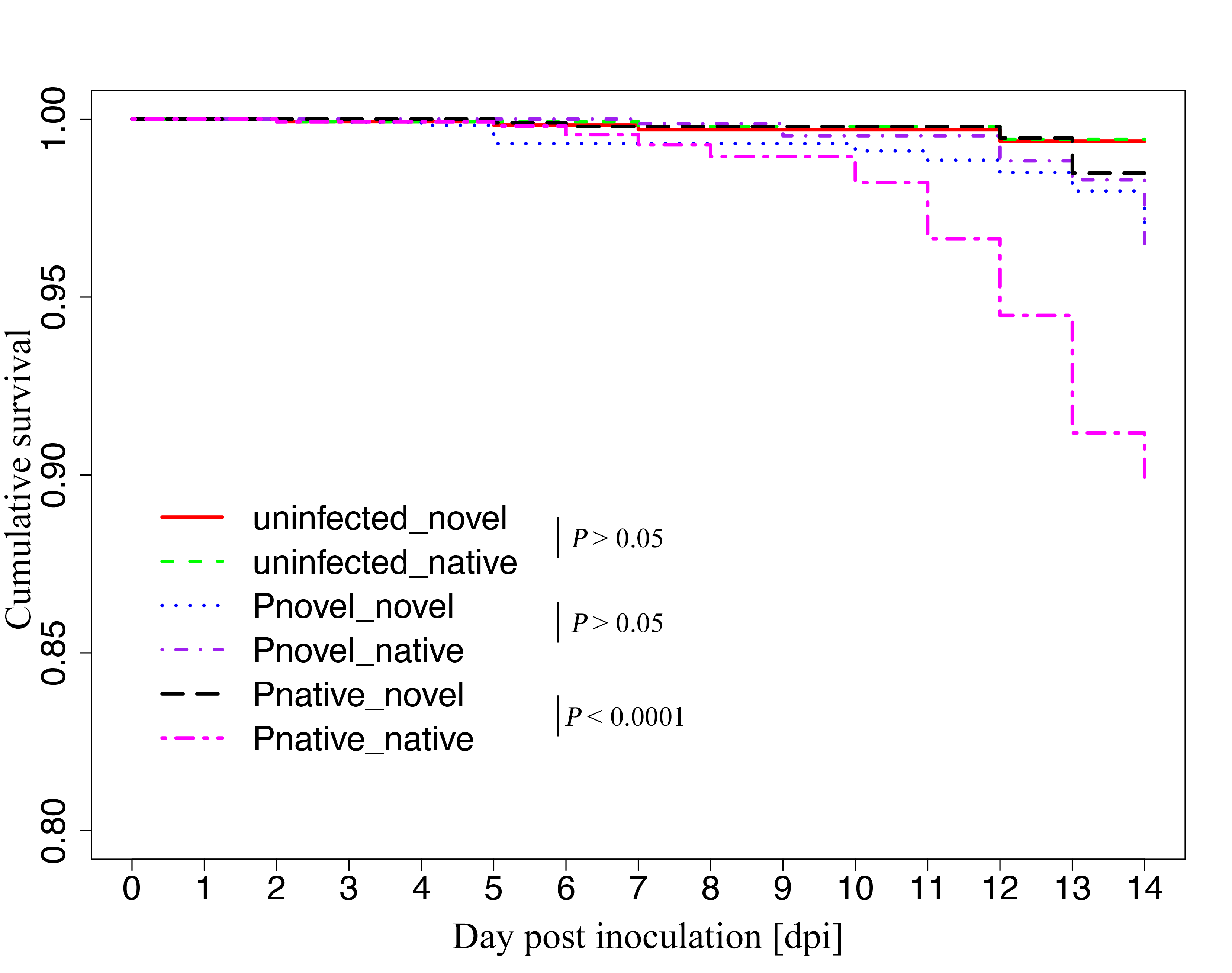


Figure S1 Cumulative survival of the bees normalized with the species variance. The uninfected bees showed the highest survival, and the Pnative_native showed the lowest survival.


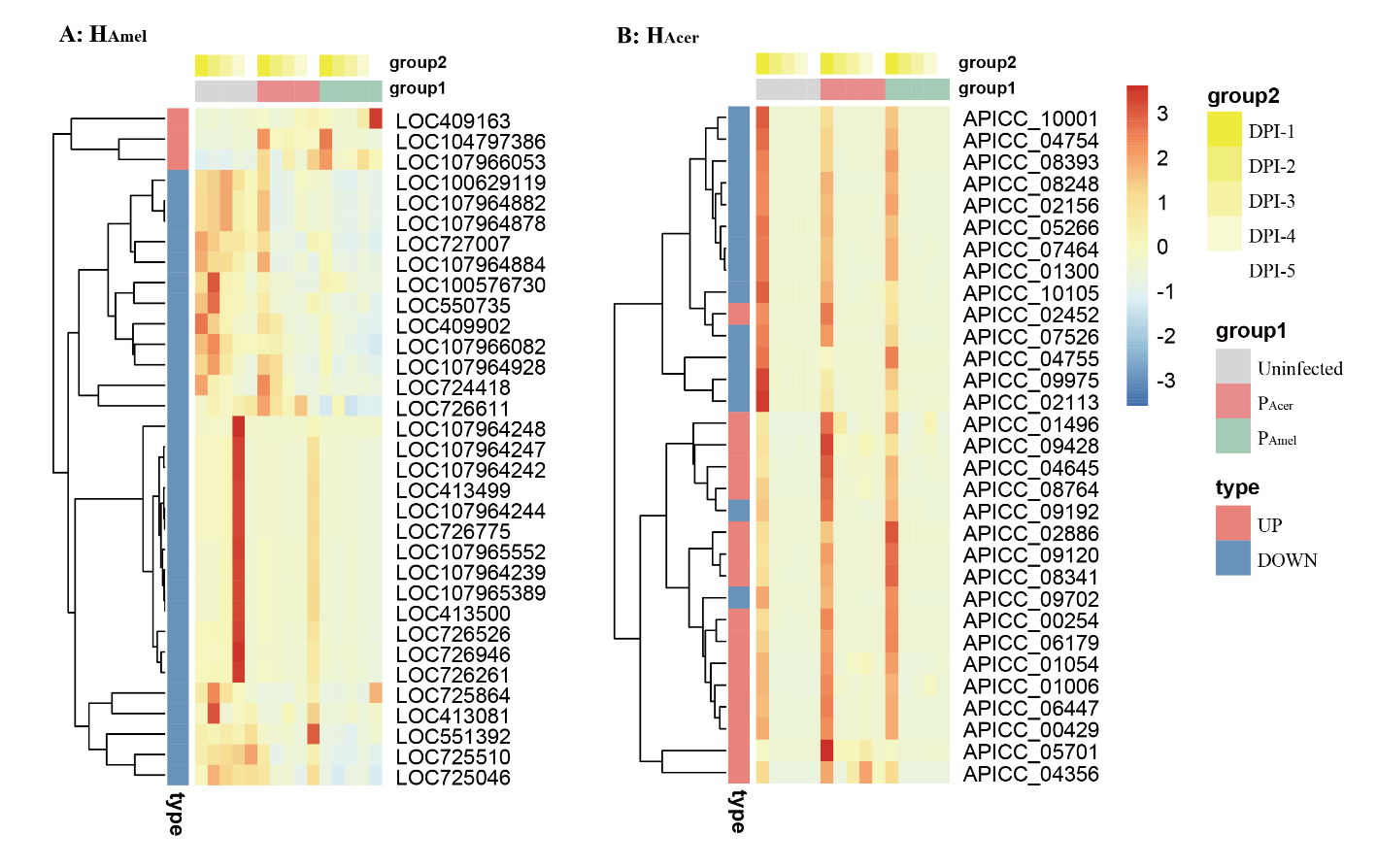


Figure S2 Heatmap of regulated Gene. (A) Genes that were consistently up or down-regulated at least four-time points in European honey bees *Apis mellifera*. (B) Genes that were consistently up or down-regulated at least four-time points in Asian honey bees *Apis cerana*.

Table S1. The experimental setup and sample size for each experimental group.

| Spore source | Inoculation | Host species | Bees per cup | Replicates | Reference |
| --- | --- | --- | --- | --- | --- |
| Purified from  *A. mellifera*  (P_Amel_) | None | *A. mellifera* | 50 | 3 | This study |
|  | None | *A. cerana* | 50 | 3 |  |
|  | 10^5^ spores | *A. mellifera* | 50 | 3 |  |
|  | 10^5^ spores | *A. cerana* | 50 | 3 |  |
| purified from  *A. cerana*  (P_Acer_) | None | *A. mellifera* | 50 | 3 | ^1^ |
|  | None | *A. cerana* | 50 | 3 |  |
|  | 10^5^ spores | *A. mellifera* | 50 | 3 |  |
|  | 10^5^ spores | *A. cerana* | 50 | 3 |  |

Table S2 Paire-wised mortality analysis among treatment groups, and the P value was adjusted for FDR.

|  | Uninfected_H_Amel_ | Uninfected_H_Acer_ | H_Amel__P_Amel_ | H_Acer__P_Amel_ |
| --- | --- | --- | --- | --- |
| Uninfected_H_Acer_ | 0.0009 |  |  |  |
| H_Amel__P_Amel_ | 0.0112 | 0.6352 |  |  |
| H_Acer__P_Amel_ | 0.0017 | 0.8785 |  |  |
| H_Amel__P_Acer_ | 0.6993 | 0.0233 | 0.06507 |  |
| H_Acer__P_Acer_ | 0.0001 | 0.0001 | 0.0001 | 0.0002 |

Table S3 P value pair-wised test of spore load among infected honey bees

|  | H_Amel__P_Amel_ | H_Acer__P_Amel_ | H_Acer__P_Acer_ |
| --- | --- | --- | --- |
| H_Acer__P_Amel_ | 0.0001 |  |  |
| H_Acer__P_Acer_ | 0.0001 | 0.3 |  |
| H_Amel__P_Acer_ | 0.9763 | 0.0001 | 0.0006 |

Table S4 A list of normalized gene count in three replicates and FDR of up-regulated parasite genes.

| 3dpi | H_Amel__P_Acer_ 1 | H_Amel__P_Acer_ 2 | H_Amel__P_Acer_ 3 | H_Amel__P_Amel_ 1 | H_Amel__P_Amel_ 2 | H_Amel__P_Amel_ 3 | FDR | up-regulation |
| --- | --- | --- | --- | --- | --- | --- | --- | --- |
| G9O61_00g014050 | 0.0 | 0.0 | 57.4 | 314.9 | 124.4 | 341.4 | 0.02520519 | P_Amel_ |
| G9O61_00g016190 | 141.2 | 96.2 | 200.9 | 0.0 | 0.0 | 0.0 | 0.02520519 | P_Acer_ |
| G9O61_00g018110 | 141.2 | 224.4 | 172.2 | 24.2 | 0.0 | 0.0 | 0.02520519 | P_Acer_ |
| G9O61_00g007160 | 329.5 | 128.2 | 200.9 | 24.2 | 31.1 | 0.0 | 0.03906782 | P_Acer_ |
| G9O61_00g007340 | 282.4 | 48.1 | 57.4 | 0.0 | 0.0 | 0.0 | 0.03906782 | P_Acer_ |
| G9O61_00g008990 | 47.1 | 16.0 | 28.7 | 145.3 | 311.1 | 409.7 | 0.03906782 | P_Amel_ |
| G9O61_00g010910 | 141.2 | 64.1 | 172.2 | 0.0 | 0.0 | 0.0 | 0.03906782 | P_Acer_ |
| G9O61_00g005410 | 0.0 | 0.0 | 0.0 | 157.4 | 93.3 | 68.3 | 0.04292675 | P_Amel_ |
| G9O61_00g011180 | 141.2 | 64.1 | 114.8 | 0.0 | 0.0 | 0.0 | 0.04292675 | P_Acer_ |
| G9O61_00g018020 | 188.3 | 128.2 | 28.7 | 0.0 | 0.0 | 0.0 | 0.04292675 | P_Acer_ |
| G9O61_00g018640 | 0.0 | 0.0 | 0.0 | 169.5 | 124.4 | 0.0 | 0.04292675 | P_Acer_ |
|  |  |  |  |  |  |  |  |  |
|  |  |  |  |  |  |  |  |  |
|  |  |  |  |  |  |  |  |  |
| 4dpi | H_Amel__P_Acer_ 1 | H_Amel__P_Acer_ 2 | H_Amel__P_Acer_ 3 | H_Amel__P_Amel_ 1 | H_Amel__P_Amel_ 2 | H_Amel__P_Amel_ 3 | FDR | up-regulation |
| G9O61_00g002870 | 785.6 | 211.1 | 155.2 | 45.2 | 145.4 | 0.0 | 8.06E-10 | P_Acer_ |
| G9O61_00g003110 | 226.6 | 20.4 | 28.7 | 17.0 | 11.2 | 0.0 | 8.31E-08 | P_Acer_ |
| G9O61_00g008140 | 20328.3 | 6008.3 | 7248.1 | 3302.7 | 4992.3 | 865.2 | 3.32E-07 | P_Acer_ |
| G9O61_00g017760 | 131.7 | 74.9 | 49.8 | 17.0 | 22.4 | 0.0 | 2.18E-05 | P_Acer_ |
| G9O61_00g013560 | 104.7 | 71.5 | 82.4 | 17.0 | 25.2 | 0.0 | 3.58E-05 | P_Acer_ |
| G9O61_00g008130 | 6977.5 | 1926.7 | 2315.8 | 1549.5 | 1597.0 | 582.4 | 4.39E-05 | P_Acer_ |
| G9O61_00g018740 | 89.6 | 20.4 | 23.0 | 0.0 | 11.2 | 0.0 | 4.39E-05 | P_Acer_ |
| G9O61_00g013530 | 180.2 | 78.3 | 105.4 | 33.9 | 30.8 | 16.6 | 0.00014 | P_Acer_ |
| G9O61_00g007620 | 41.0 | 85.1 | 65.1 | 19.8 | 8.4 | 0.0 | 0.00015861 | P_Acer_ |
| G9O61_00g002910 | 198.6 | 20.4 | 40.2 | 22.6 | 16.8 | 16.6 | 0.00024201 | P_Acer_ |
| G9O61_00g007050 | 14.0 | 6.8 | 11.5 | 33.9 | 55.9 | 99.8 | 0.00033813 | P_Amel_ |
| G9O61_00g014590 | 131.7 | 81.7 | 46.0 | 17.0 | 0.0 | 49.9 | 0.00068597 | P_Acer_ |
| G9O61_00g017460 | 6351.6 | 2171.8 | 2208.5 | 1753.1 | 1901.8 | 482.5 | 0.00068597 | P_Acer_ |
| G9O61_00g020820 | 51.8 | 13.6 | 42.1 | 5.7 | 5.6 | 0.0 | 0.00079955 | P_Acer_ |
| G9O61_00g022020 | 51.8 | 13.6 | 42.1 | 5.7 | 5.6 | 0.0 | 0.00079955 | P_Acer_ |
| G9O61_00g015320 | 30.2 | 64.7 | 46.0 | 118.8 | 125.9 | 191.3 | 0.00255527 | P_Amel_ |
| G9O61_00g021890 | 360.4 | 78.3 | 205.0 | 73.5 | 50.3 | 116.5 | 0.00434521 | P_Acer_ |
| G9O61_00g016260 | 88.5 | 27.2 | 34.5 | 8.5 | 8.4 | 16.6 | 0.00451742 | P_Acer_ |
| G9O61_00g019740 | 50.7 | 74.9 | 72.8 | 169.7 | 167.8 | 199.7 | 0.00635098 | P_Amel_ |
| G9O61_00g010700 | 101.4 | 61.3 | 17.2 | 19.8 | 8.4 | 16.6 | 0.00732356 | P_Acer_ |
| G9O61_00g022730 | 32.4 | 6.8 | 7.7 | 0.0 | 0.0 | 0.0 | 0.0091431 | P_Acer_ |
| G9O61_00g020790 | 18.3 | 71.5 | 38.3 | 8.5 | 5.6 | 8.3 | 0.01076239 | P_Acer_ |
| G9O61_00g005250 | 835.2 | 343.8 | 273.9 | 243.2 | 218.2 | 166.4 | 0.01494163 | P_Acer_ |
| G9O61_00g010150 | 21.6 | 27.2 | 26.8 | 8.5 | 0.0 | 0.0 | 0.0150468 | P_Acer_ |
| G9O61_00g021650 | 50.7 | 74.9 | 76.6 | 164.0 | 165.0 | 183.0 | 0.01613343 | P_Amel_ |
| G9O61_00g016140 | 19.4 | 13.6 | 23.0 | 70.7 | 28.0 | 99.8 | 0.02082087 | P_Amel_ |
| G9O61_00g018640 | 3.2 | 0.0 | 15.3 | 33.9 | 53.1 | 0.0 | 0.02169993 | P_Amel_ |
| G9O61_00g001240 | 31.3 | 27.2 | 32.6 | 104.6 | 64.3 | 83.2 | 0.02386671 | P_Amel_ |
| G9O61_00g006350 | 66.9 | 30.6 | 21.1 | 17.0 | 11.2 | 0.0 | 0.02386671 | P_Acer_ |
| G9O61_00g007110 | 4.3 | 0.0 | 11.5 | 8.5 | 11.2 | 99.8 | 0.02386671 | P_Amel_ |
| G9O61_00g011470 | 265.5 | 102.1 | 99.6 | 79.2 | 89.5 | 16.6 | 0.0240823 | P_Acer_ |
| G9O61_00g012660 | 15.1 | 40.8 | 19.2 | 8.5 | 0.0 | 0.0 | 0.0240823 | P_Acer_ |
| G9O61_00g014630 | 23.7 | 20.4 | 17.2 | 56.6 | 22.4 | 141.4 | 0.0240823 | P_Amel_ |
| G9O61_00g005110 | 23.7 | 3.4 | 21.1 | 2.8 | 0.0 | 0.0 | 0.02737697 | P_Acer_ |
| G9O61_00g011090 | 30.2 | 27.2 | 26.8 | 90.5 | 64.3 | 83.2 | 0.02737697 | P_Amel_ |
| G9O61_00g021160 | 12.9 | 20.4 | 19.2 | 11.3 | 39.2 | 149.8 | 0.02737697 | P_Amel_ |
| G9O61_00g001470 | 32.4 | 27.2 | 32.6 | 104.6 | 64.3 | 83.2 | 0.02790996 | P_Amel_ |
| G9O61_00g015700 | 147.8 | 54.5 | 72.8 | 39.6 | 44.7 | 16.6 | 0.03044956 | P_Acer_ |
| G9O61_00g006280 | 271.9 | 153.2 | 206.9 | 96.1 | 156.6 | 16.6 | 0.03387642 | P_Acer_ |
| G9O61_00g008320 | 200.7 | 74.9 | 61.3 | 62.2 | 72.7 | 0.0 | 0.04037523 | P_Acer_ |
| G9O61_00g019860 | 49.6 | 10.2 | 28.7 | 5.7 | 0.0 | 16.6 | 0.04310919 | P_Acer_ |
| G9O61_00g003150 | 229.9 | 61.3 | 76.6 | 67.9 | 50.3 | 33.3 | 0.04345143 | P_Acer_ |
| G9O61_00g003140 | 9.7 | 13.6 | 7.7 | 0.0 | 0.0 | 0.0 | 0.04394313 | P_Acer_ |
| G9O61_00g009450 | 9.7 | 23.8 | 1.9 | 0.0 | 0.0 | 0.0 | 0.04394313 | P_Acer_ |
| G9O61_00g017170 | 595.7 | 394.9 | 254.8 | 271.5 | 170.6 | 149.8 | 0.04692882 | P_Acer_ |
| G9O61_00g018900 | 24.8 | 40.8 | 7.7 | 0.0 | 11.2 | 0.0 | 0.04692882 | P_Acer_ |
| G9O61_00g020150 | 11.9 | 27.2 | 32.6 | 2.8 | 8.4 | 0.0 | 0.04692882 | P_Acer_ |
| G9O61_00g003850 | 228.8 | 61.3 | 72.8 | 67.9 | 50.3 | 33.3 | 0.04743578 | P_Acer_ |
|  |  |  |  |  |  |  |  |  |
| 5dpi | PnativeHnovel1 | PnativeHnovel2 | PnativeHnovel3 | PnovelHnovel1 | PnovelHnovel2 | PnovelHnovel3 | FDR | up-regulation |
| G9O61_00g004240 | 1.4 | 2.2 | 0.0 | 8.9 | 5.0 | 26.8 | 0.02976872 | P_Amel_ |
| G9O61_00g013420 | 26.1 | 10.9 | 6.5 | 48.7 | 39.9 | 58.1 | 0.04159174 | P_Amel_ |
| G9O61_00g016880 | 56.4 | 25.2 | 129.1 | 25.5 | 37.4 | 4.5 | 0.04159174 | P_Acer_ |
| G9O61_00g000020 | 162.1 | 75.5 | 159.3 | 73.1 | 44.9 | 31.3 | 0.04613185 | P_Acer_ |
| G9O61_00g008260 | 0.0 | 0.0 | 0.0 | 4.8 | 15.0 | 4.5 | 0.04613185 | P_Amel_ |
| G9O61_00g018460 | 150.5 | 71.1 | 159.3 | 69.5 | 42.4 | 33.5 | 0.04613185 | P_Acer_ |
| G9O61_00g022710 | 46.3 | 63.4 | 53.8 | 29.7 | 19.9 | 4.5 | 0.04613185 | P_Acer_ |
